# Noncontiguous Finished Genome Sequence of *Staphylococcus aureus* KLT6, a Staphylococcal Enterotoxin B-Positive Strain Involved in a Food Poisoning Outbreak in Switzerland

**DOI:** 10.1128/genomeA.00214-13

**Published:** 2013-05-23

**Authors:** Raquel Tobes, Marina Manrique, Marta Brozynska, Roger Stephan, Eduardo Pareja, Sophia Johler

**Affiliations:** Oh No Sequences! Research group at Era7 Bioinformatics, Granada, Spaina; Institute for Food Safety and Hygiene, Vetsuisse Faculty, University of Zurich, Zurich, Switzerlandb

## Abstract

We present the first complete genome sequence of a *Staphylococcus aureus* strain assigned to clonal complex 12. The strain was isolated in a food poisoning outbreak due to contaminated potato salad in Switzerland in 2009, and it produces staphylococcal enterotoxin B.

## GENOME ANNOUNCEMENT

*Staphylococcus aureus* not only represents a commensal that colonizes the nares of 20 to 30% of the global population (1), but it also causes severe infections, toxinoses, and life-threatening illnesses. Staphylococcal food poisoning is one of the most prevalent causes of food-borne intoxication worldwide. Shortly after intake of staphylococcal enterotoxins, patients exhibit violent emesis.

On 7 October 2009, ca. 400 people gathered for a yodeling festival in Münchwilen (Switzerland). Upon ingestion of contaminated potato salad, 30 participants suffered from acute vomiting and diarrhea. The outbreak was traced back to a staphylococcal enterotoxin B (SEB)-producing *S. aureus* strain that was designated strain KLT6 and was assigned to CC12 and *spa* type t160 (2).

We determined the genome sequence of KLT6 by combining optical mapping, long CCS (circular consensus sequencing) PacBio reads, and short Illumina reads.

A paired-end library of the KLT6 genome was created and sequenced using the Illumina HiSeq 2000 sequencer (GATC Biotech AG, Konstanz, Germany). Unspecified nucleotides (N) were removed and the 199,617,120 50-bp Illumina reads were *de novo* assembled using Velvet (3), resulting in 152 contigs. Optical mapping after digestion of KLT6 with NcoI and generation of PacBio reads using single-molecule real-time sequencing (SMRT) were outsourced to OpGen (Gaithersburg, MD) and Expression Analysis (Durham, NC). The assembly was refined to 31 contigs without any N, a maximum length contig of 1,275,455 bp and N_60_ of 313,040 bp, and one scaffold. The scaffolding was carried out using the optical map and the CCS PacBio reads (coverage, ~1×). Mummer and BLAST results of the alignment of the PacBio CCS and PacBio standard long reads to the contigs obtained with Velvet were used for scaffolding. A custom program based on graph databases was developed for connecting contigs, adding new sequence data from CCS at gaps when needed. Alignments to the regions of reference genomes (especially *S. aureus* NCTC 8325) exhibiting *in silico* restriction maps identical to those of KLT6 were also used in the final refinement of the assembly. RNA operons that appeared to be collapsed in the preliminary Velvet assembly were manually reconstructed by analyzing regions with significantly higher coverage than their neighboring sequences and by searching for compatible reads. The genome was annotated with BG7 (4) and contigs were connected when the joining of partial coding sequences located at the end of contigs could complete a specific protein.

One contiguous finished scaffold represents the complete circular KLT6 chromosome, consisting of 2,705,935 bp with a G+C content of 32.79%. The genome sequence of KLT6 contains 2,470 protein-coding genes, four complete 16S-5S-23S operons and 18 tRNA genes, ten noncoding RNAs, including an RNAIII regulatory transcript containing the delta hemolysin structural gene, eight riboswitches, a glucosamine-6-phosphate-activated ribozyme, and genes similar to bacteriophage phi-X174. Contigs KLT6000017 and KLT6000020 include 23 genes, many of them similar to the genes of plasmid pUSA300HOUMS in *S. aureus* USA300 TCH959.

### Nucleotide sequence accession numbers.

The sequence and annotation data of the *S. aureus* KLT6 genome were deposited in the GenBank database. This Whole-Genome Shotgun project was deposited at DDBJ/EMBL/GenBank under the accession no. APFH00000000. The version described in this paper is the first version, accession no. APFH01000000.
